# Periconceptional and Gestational Exposure to Antibiotics and Childhood Asthma

**DOI:** 10.1371/journal.pone.0140443

**Published:** 2015-10-21

**Authors:** Shuyuan Chu, Hongping Yu, Yan Chen, Qian Chen, Bin Wang, Jun Zhang

**Affiliations:** 1 MOE-Shanghai Key Laboratory of Children’s Environmental Health, Xinhua Hospital, Shanghai Jiao Tong University School of Medicine, Shanghai, 200092, China; 2 School of Public Health, Guilin Medical University, Guilin, 541004, China; 3 Collaborative Innovation Center of Systems Biomedicine, Shanghai Jiao Tong University School of Medicine, Shanghai, 200025, China; University Children's Hospital Basel, SWITZERLAND

## Abstract

**Background:**

Previous studies suggest that maternal antibiotics exposure during pregnancy may increase the risk of childhood asthma, but the results were inconsistent. Furthermore, most studies did not examine periconception period as an exposure window. We aim to assess the associations between maternal exposure to specific antibiotics before and during pregnancy and the risk of asthma in early childhood.

**Methods:**

Data from the Collaborative Perinatal Project were used. Maternal exposure to antibiotics before and during pregnancy was recorded at each prenatal visit. A total of 39,907 singleton children were followed up to 7 years of age. Multilevel multiple logistic regression models were used to control for potential confounders and account for multiple pregnancies per woman.

**Results:**

Maternal use of penicillin or chloramphenicol was associated with an increased risk of asthma in the offspring (adjusted odds ratio = 1.21, 95% confidence interval 1.08–1.36 for penicillin; 1.72 [1.14–2.59] for chloramphenicol). The risk was significantly increased if penicillin or chloramphenicol was used in the 1st trimester (1.09 [1.04–1.13] for penicillin and 1.23 [1.01–1.51] for chloramphenicol).

**Conclusion:**

Maternal exposure to certain antibiotics is associated with childhood asthma by 7 years of age. Early pregnancy may be a sensitive window.

## Introduction

Asthma imposes a great health burden globally. It was estimated that about 300 million people were affected in 2011[1], and 250,000–345,000 people die from asthma each year [2]. From public health perspectives, more attention is needed to the etiology of asthma, especially in children, for whom the incidence of asthma was more than three times of that in adults [3].

Previous studies showed that maternal antibiotics exposure during pregnancy was associated with an increased risk of childhood asthma but the results were inconsistent. [4–9]. For example, Stensballe et al. reported that maternal exposure to different types of antibiotics in the third trimester was associated with elevated childhood asthma incidence [8], but McKeever et al. [7] failed to find any significant associations. Moreover, none of the previous studies examined the periconceptional window, which is a crucial period for embryo development [10]. Therefore, we conducted the present study to assess the associations between maternal exposure to different types of antibiotics before and during pregnancy and childhood asthma in the Collaborative Perinatal Project (CPP), a large prospective birth cohort study in the United States in 1959–1976.

## Methods

### Study population

**Fig 1:**
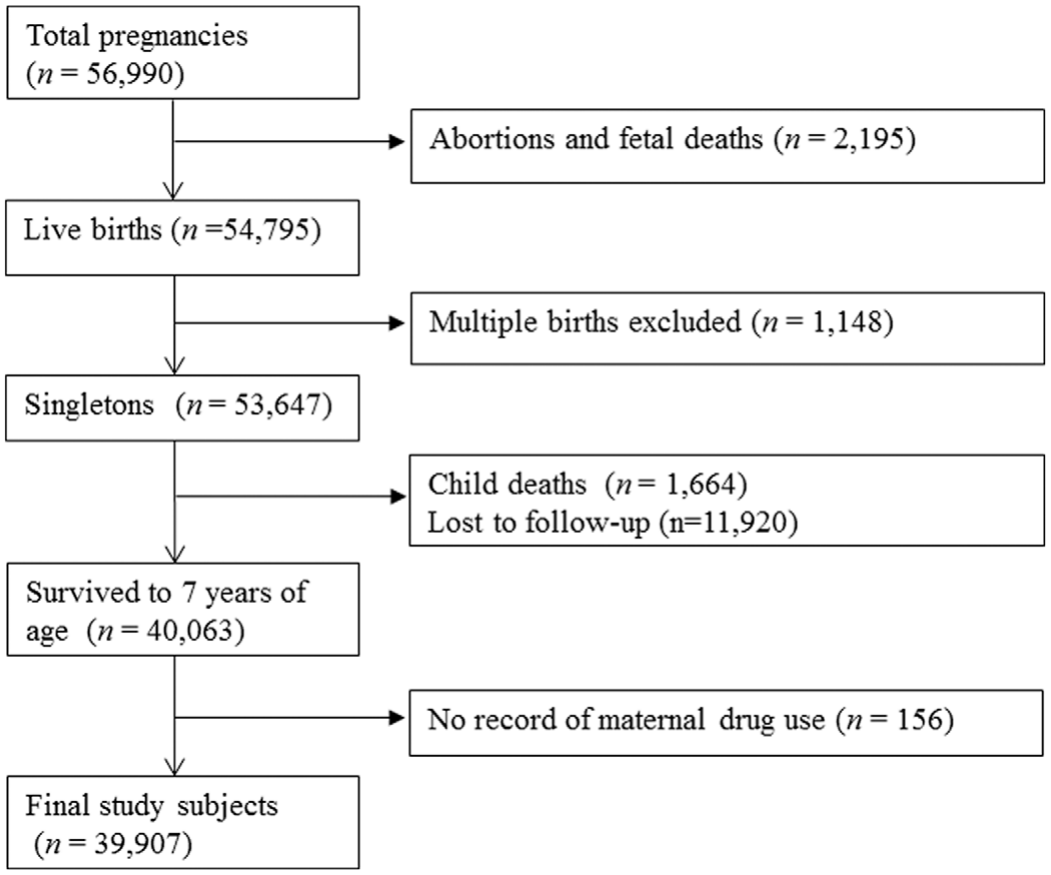
Study population flow chart, Collaborative Perinatal Project, United States, 1959–1965.

A detailed description of the study population and method has been provided elsewhere [11]. Briefly, 46,021 women with 56,990 pregnancies were enrolled at 12 sites in the United States from 1959 to 1965. These women were routinely interviewed during pregnancy and the newborns were followed up. Approximately 80 percent of the children were followed up to the age of seven to eight years. Physical examinations of neurological, neurosensory, cognitive development, general health and physical growth were taken at eight months, one, three, four, seven, and eight years of age in children [12]. Among 53,647 singleton births, 40,063 were followed until at least 7 years of age. The CPP was carried out by researchers who conducted the original study from 1959–1976.We excluded 156 children without a record of maternal periconceptional medication, leaving 39,907 children for the final analysis (Fig 1).

### Exposure to antibiotics

At the enrollment and each prenatal visit, women were asked regarding medication before and during pregnancy as previously described [13,14]. The name of medicine and the time of medicine exposure were recorded. The maternal antibiotic exposure was defined as the mother taking antibiotics by oral or injection during 4 weeks prior to the last menstrual period (LMP)or at any month during pregnancy. We separated the exposure windows into periconception (within 28 days prior to LMP), 1^st^ (day 1 after LMP to day 91), 2^nd^ (day 92 to 189), and 3^rd^ trimesters (day 190 to delivery).

### Diagnosis of asthma

Medical records of each child from birth through the age of 7 years were reviewed by pediatricians. The diagnosis of asthma was made as “suspected”, “definite,” or “history only”. The present study used “definite” asthma by 7 years of age as the outcome. The “definite asthma” of children was identified by the relevant code from the World Health Organization International Classification of Diseases (ICD) in the revisions, including 241 in the 7th revision in 1958, and 493 in the 8th revision in 1968.This classification scheme was consistent with previous studies using the data from CPP [15,16].

### Covariates

The following prenatal factors were included as potential confounders for the associations between maternal exposure to antibiotics and childhood asthma [17], maternal age at delivery (<20, 20–29, 30–39, or≥40 years), married at pregnancy (no/yes); race (white, black, or other); educational level (<9, 10–12, or >12 years); number of previous births (0, 1, or≥2); smoking during pregnancy (0, 1–9, or≥10 cigarettes per day), maternal asthma history (no/yes), and maternal drug allergy history (no/yes). If a covariant changed the association between exposure and outcome by 10% or more in a logistic regression model, we considered this variable as a potential confounder and controlled for it.

### Statistical analysis

Chi-square test was used to assess the differences of maternal and children’s characteristics at baseline in children with and without asthma. Because some women gave more than one birth in the CPP, the associations between maternal exposure to antibiotics and the risk of childhood asthma were explored with multilevel multiple logistic regression models, accounting for the correlation between births from the same mother. The results were presented as odds ratios (OR) and 95% confidence intervals (CI). All statistical analysis was performed using SAS 9.2 (SAS Institute, Inc., Cary,North Carolina).

## Results

The prevalence of asthma was 5.5% (2,201/39,907) in this population. Baseline characteristics of these children are shown by asthma status in Table 1. No significant differences were observed in maternal age at pregnancy, maternal educational level, maternal smoking during pregnancy, and delivery mode between asthma and non-asthma groups. However, the asthmatic children had a higher proportion of unmarried, asthmatic mothers, mothers with drug allergy history, male infants and black race (all p<0.05).

**Table 1:** Maternal and Child Characteristics by Asthma Status in the Collaborative Perinatal Project, United States, 1959–1965.

Characteristic	Nonasthma (n = 37706)		Asthma (n = 2201)		P value ^a^
	No.	%	No.	%	
Maternal race					<0.001
White	17598	46.7	849	38.6	
Black	18587	49.3	1222	55.5	
Other	1507	4.0	130	5.9	
Maternal age, (years)					0.370
<20	8830	23.4	535	24.3	
20–29	21348	56.6	1204	54.7	
30–39	6837	18.1	420	19.1	
≥40	691	1.8	42	1.9	
Married at pregnancy	29202	77.4	1644	74.7	0.003
Maternal education (years)					0.180
≤9	10469	27.8	652	29.6	
10–12	22403	59.4	1276	58.0	
>12	4266	11.3	243	11.0	
Smoking during pregnancy					0.187
Nonsmoker (0 cig./day)	19967	53.0	1146	52.1	
Light smoker (1–9 cig./day)	6576	17.4	419	19.0	
Heavy smoker (≥10 cig./day)	10872	28.3	628	28.5	
Maternal asthma	844	2.2	152	6.9	<0.001
Maternal drug allergy	2072	5.5	159	7.2	0.001
Parity					0.275
0	738	2.0	52	2.4	
1	8390	22.3	492	22.4	
≥2	18293	48.5	1031	46.8	
Mode of delivery					0.425
Vaginal	34710	92.1	2015	91.5	
Assisted vaginal	843	2.2	58	2.6	
Cesarean	2057	5.5	125	5.7	
Birthweight, g					0.024
<2500	3476	9.2	244	11.1	
2500–2999	9313	24.7	555	25.2	
3000–3499	15028	39.9	869	39.5	
3500–3999	7841	20.8	420	19.1	
≥4000	2034	5.4	113	5.1	
Preterm birth(<37 weeks)	5678	15.1	408	18.5	<0.001
Male sex	18809	49.9	1382	62.8	<0.001
5-minute Apgar score 0–6	1349	3.6	88	4.0	0.303

^a^: P values were determined by χ^2^ test.

A total of 10,534 (26.4%) mothers were treated with antibiotics one month prior to LMP or during pregnancy. The vast majority of them used one type of antibiotics (22.5%). Penicillin was the most commonly used antibiotic (15.6% of all mothers) followed by sulfonamides and trimethoprim (10.1% of all mothers). Most mothers received antibiotic treatment in the second (12.8% of all mothers) or third (12.7% of all mothers) trimesters.

Table 2 shows the associations between maternal exposure to various antibiotics before and during pregnancy and asthma in the offspring. Maternal use of penicillin or chloramphenicol was associated with a significantly increased risk of childhood asthma after adjusting for potential confounders (adjusted OR = 1.21 [95% CI1.08–1.36] for penicillins; adjusted OR = 1.72 [95% CI1.14–2.59] for chloramphenicols). We further separated the results by the time of medication. Penicillins or chloramphenicols use in first trimester was significantly associated with childhood asthma (adjusted OR = 1.09 [1.04–1.13] and1.23 [1.01–1.51], respectively).When we further controlled for low birthweight and preterm birth as potential confounders in the multilevel logistic regression models, the results didn’t change.

**Table 2:** Adjusted and unadjusted risk of childhood asthma exposed to maternal antibiotic use during 4 weeks before LMP and pregnancy and followed to 7 years of age.

Antibiotics exposure	Number of mothers using antibiotics	Unadjusted estimates			Adjusted estimates ^a^		
		OR	95%CI	P value	OR	95%CI	P value
**Penicillins**							
total	6208	1.25	1.11–1.40	<0.001	1.21	1.09–1.36	0.001
4 weeks before LMP	751	1.07	0.99–1.15	0.069	1.06	0.99–1.14	0.100
1st trimester	2305	1.09	1.05–1.14	<0.001	1.09	1.04–1.13	<0.001
2nd trimester	2619	1.03	0.99–1.07	0.209	1.02	0.98–1.06	0.376
3rd trimester	1899	1.01	0.97–1.07	0.583	1.01	0.96–1.07	0.606
**Sulfonamides and trimethoprim**							
total	4044	1.03	0.89–1.19	0.688	0.98	0.85–1.13	0.756
4 weeks before LMP	165	1.08	0.93–1.25	0.339	1.07	0.92–1.25	0.354
1st trimester	676	1.03	0.95–1.11	0.529	1.02	0.94–1.10	0.707
2nd trimester	1687	1.05	1.00–1.11	0.044	1.04	0.99–1.09	0.131
3rd trimester	2264	0.98	0.94–1.03	0.459	0.97	0.92–1.02	0.184
**Chloramphenicols**							
total	290	1.77	1.18–2.65	0.006	1.72	1.14–2.59	0.009
4 weeks before LMP	15	1.05	0.63–1.76	0.845	1.05	0.62–1.76	0.857
1st trimester	58	1.24	1.01–1.52	0.038	1.23	1.01–1.51	0.044
2nd trimester	128	1.16	0.99–1.34	0.062	1.13	0.97–1.32	0.107
3rd trimester	125	1.11	0.94–1.30	0.232	1.11	0.94–1.31	0.223
**Tetracycline**							
total	549	1.25	0.99–1.57	0.067	1.20	0.95–1.52	0.133
4 weeks before LMP	19	1.19	0.82–1.73	0.354	1.21	0.83–1.76	0.314
1st trimester	77	0.82	0.58–1.17	0.281	0.84	0.59–1.20	0.341
2nd trimester	227	1.03	0.90–1.18	0.660	1.02	0.89–1.17	0.814
3rd trimester	260	1.09	0.97–1.23	0.130	1.07	0.95–1.20	0.280
**Nitrofurans**							
total	385	1.21	0.84–1.73	0.307	1.12	0.78–1.61	0.552
4 weeks before LMP	9	^b^	^b^		^b^	^b^	
1st trimester	29	1.18	0.87–1.61	0.278	1.21	0.89–1.65	0.220
2nd trimester	142	1.01	0.84–1.21	0.948	0.98	0.81–1.17	0.789
3rd trimester	248	1.04	0.91–1.18	0.550	1.02	0.90–1.16	0.779
**Otherantibiotics**							
total	727	1.26	1.00–1.59	0.047	1.21	0.96–1.53	0.105
4 weeks before LMP	90	1.00	0.80–1.26	0.973	0.99	0.79–1.24	0.915
1st trimester	228	1.03	0.90–1.18	0.669	1.01	0.88–1.16	0.930
2nd trimester	302	0.97	0.85–1.11	0.683	0.97	0.85–1.11	0.695
3rd trimester	280	1.01	0.89–1.15	0.867	1.01	0.89–1.15	0.883

^a^: adjusted for maternal age at delivery, marital status at pregnancy, race, educational level, parity, smoking during pregnancy, maternal asthma history, and maternal history of drug allergy.

^b^: The precise value can’t becalculated, because the sample size was too small to analyze.

LMP: last menstrual period

A sensitivity analysis was conducted by excluding women who reported having exposed to two or more types of antibiotics before or during pregnancy. The results remained virtually the same (not shown). When we stratified the results by the time of medication, again the results were very similar (S1 Table).

## Discussion

In this prospective cohort study, we found that use of penicillins or chloramphenicols during early pregnancy was associated with an increased risk of asthma in offspring after controlling for potential confounders. These findings suggest that early life exposure to certain antibiotics might increase the risk of childhood asthma.

Our finding is consistent with two Danish longitudinal cohort studies, in which if mothers used antibiotics at any time during pregnancy, the children had an increased risk of asthma hospitalization (hazard ratio 1.17 [1.00–1.36]), and inhaled corticosteroids (1.18 [1.10–1.27]) [8]. However, a recent Swedish study using sibling analysis suggested that previous positive findings may have been due to confounding by indication or uncontrolled genetic and environmental confounders [18]. Although the sibling analysis may have adjusted residual familial factors better, the exposure combined all types of antibiotics in that study [18]. Our study showed when all antibiotics were combined, the significant association found in penicillin was reduced, indicating that the association may be antibiotic-specific.

Some old antibiotics are no longer being used in contemporary practice. However, our findings raised several questions that may still have important clinical and pharmacodevelopmental implications. In pregnancy, different antibiotics have specific effect on changing maternal microbiota [19,20]. The latter could be transmitted to the fetus not only during delivery [21], but during pregnancy as well [21–23]. The placental microbiome produces metabolic and immune factors, and generates a balanced mucosal immune system [24]. When microbial exposure of fetus was disturbed, the risk of atopic immune responses and asthma is increased [25]. Thus, we speculate that the effect on asthma risk may be related to the antibacterial spectrum of penicillins or chloramphenicols, both of which target gram positive bacteria. Detailed types of microbiota and their explicit mechanism need to be further elucidated in future epidemiologic studies and *in vitro* experiments.

Our study also found that the early pregnancy may be a sensitive window for the exposure to penicillins or chloramphenicols. The early pregnancy is an important time for fetal immune development [26]. Even at the 12 weeks of gestation, T cell has begun to develop [26]. Furthermore, the sensitive window may also depend on the type of antibiotics. More research is needed. Since in CPP data, women were asked about their health condition 4 weeks before LMP, including medication, we used 4-week timeframe and were unable to restrict our analysis to exposure periods closer to the time of actual conception.

We acknowledge that the diagnostic criteria for asthma may have changed since 1960s. The asthma diagnosis then was based on notion that asthma is “a disease of the respiratory passages characterized by dyspnea of an obstructive type which is predominantly expiratory, reversible at least partially, and of varying severity and duration” [27]. Even today, asthma diagnosis in young children is still made based largely on symptom patterns and on a careful clinical assessment of family history and physical findings according to the Global Initiative for Asthma works [28,29]. This is due to the fact that lung function measures in children may not be reliable, even in children of 7 years [30]. In our study, we included only “definite asthma” confirmed by pediatricians and excluded suspected asthma. The prevalence of asthma was 5.5% at that time, suggesting that misclassification of asthma may not be a serious issue in our study.

It may be questioned whether the association between maternal antibiotic use before and during pregnancy and childhood asthma was due to confounding by indication or genetics, i.e., women who used antibiotics tended to have an elevated risk of asthma-related diseases. This tendency may pass down to their children. Thus, it would be the genetic susceptibility to asthma rather than antibiotic use *per se* that has caused this association [18]. Unfortunately, CPP did not collect information on indication for maternal antibiotic use. However, only maternal urinary tract infections during pregnancy were found to increase the risk of asthma in children in previous studies [31], whereas penicillins or chloramphenicols weren’t indicated to use in urinary tract infections. Therefore, the association between maternal exposure to penicillins or chloramphenicols and childhood asthma may not be confounded by maternal infections that led to penicillins or chloramphenicols use. Moreover, our study shows that even after we adjusted for history of maternal asthma and allergy, exposure to certain drugs, namely penicillins and chloramphenicols, during early pregnancy was associated with child asthma.

In addition, over 11,000 children were lost to follow-up and, therefore, often did not have asthma diagnosis. But the prevalence of antibiotic exposure or asthma history of mothers was similar between those lost and followed-up. For example, the prevalence of antibiotic exposure was 26.4% vs 27.7% for mothers included vs mothers with loss-to-follow-up, respectively. Likewise, the prevalence of maternal asthma history was 4.7% vs 5.3%, respectively. These differences appeared small and were unlikely to bias the results substantially.

In conclusion, maternal exposure to penicillins or chloramphenicols during pregnancy is associated with childhood asthma by 7 years of age. Early pregnancy may be a sensitive window. Our findings not only confirmed previous reports that maternal exposure to antibiotics enhances the risk of childhood asthma, but also showed that this association may be antibiotic-specific.

## Supporting Information

S1 Table Adjusted and unadjusted risk of childhood asthma and maternal antibiotic use stratified by the time of medication. (DOCX)

## Abbreviations

CI: confidence intervals
CPP: Collaborative Perinatal Project
LMP: last menstrual period
OR: odds ratio
