# Supplementary material for: Periconceptional and Gestational Exposure to Antibiotics and Childhood Asthma
Source: PLoS One. 2015 Oct 21;10(10):e0140443. doi: 10.1371/journal.pone.0140443 (PMC4619063; doi:10.1371/journal.pone.0140443)
Supplement: S1 Table — (DOCX) [file pone.0140443.s001.docx]

Table S1. Adjusted and unadjusted risk of childhood asthma and maternal antibiotic use stratified by the time of medication.

|  |  | **Unadjusted estimates** | | | **Adjusted estimates ^a^** | | |
| --- | --- | --- | --- | --- | --- | --- | --- |
| **Time of antibiotics exposure** | **Number of mothers using antibiotics** | **OR** | **95%CI** | **P value** | **OR** | **95%CI** | **P value** |
| total | 10534 | 1.17 | 1.07-1.29 | 0.001 | 1.13 | 1.02-1.24 | 0. 015 |
| 4 weeks before LMP | 1005 | 1.07 | 1.01-1.14 | 0.033 | 1.07 | 1.00-1.13 | 0.049 |
| 1st trimester | 3128 | 1.08 | 1.04-1.12 | <0.001 | 1.08 | 1.04-1.11 | <0.001 |
| 2nd trimester | 4645 | 1.03 | 1.00-1.06 | 0.072 | 1.02 | 0.99-1.06 | 0.224 |
| 3rd trimester | 4675 | 1.02 | 0.98-1.05 | 0.371 | 1.01 | 0.97-1.04 | 0.757 |

a: adjusted for maternal age at delivery, marital status at pregnancy, race, educational level, parity, smoking during pregnancy, maternal asthma history, and maternal history of drug allergy.

LMP: last menstrual period
